# Targeting and tracing of specific DNA sequences with dTALEs in living cells

**DOI:** 10.1093/nar/gkt1348

**Published:** 2013-12-25

**Authors:** Katharina Thanisch, Katrin Schneider, Robert Morbitzer, Irina Solovei, Thomas Lahaye, Sebastian Bultmann, Heinrich Leonhardt

**Affiliations:** ^1^Department of Biology II, Humanbiology and Bioimaging, Center for Integrated Protein Science Munich (CIPSM), Ludwig Maximilians University Munich, 82152 Planegg-Martinsried, Germany and ^2^Department of Biology I, Genetics, Ludwig Maximilians University Munich, 82152 Planegg-Martinsried, Germany

## Abstract

Epigenetic regulation of gene expression involves, besides DNA and histone modifications, the relative positioning of DNA sequences within the nucleus. To trace specific DNA sequences in living cells, we used programmable sequence-specific DNA binding of designer transcription activator-like effectors (dTALEs). We designed a recombinant dTALE (msTALE) with variable repeat domains to specifically bind a 19-bp target sequence of major satellite DNA. The msTALE was fused with green fluorescent protein (GFP) and stably expressed in mouse embryonic stem cells. Hybridization with a major satellite probe (3D-fluorescent *in situ* hybridization) and co-staining for known cellular structures confirmed *in vivo* binding of the GFP-msTALE to major satellite DNA present at nuclear chromocenters. Dual tracing of major satellite DNA and the replication machinery throughout S-phase showed co-localization during mid to late S-phase, directly demonstrating the late replication timing of major satellite DNA. Fluorescence bleaching experiments indicated a relatively stable but still dynamic binding, with mean residence times in the range of minutes. Fluorescently labeled dTALEs open new perspectives to target and trace DNA sequences and to monitor dynamic changes in subnuclear positioning as well as interactions with functional nuclear structures during cell cycle progression and cellular differentiation.

## INTRODUCTION

Covalent DNA and histone modifications play a key role in epigenetic gene regulation and have been intensively investigated over the past decades. While there is no doubt that higher order chromatin structures and nuclear genome organization also play important roles, they are far less amenable to systematic analysis due to their transient and fragile nature that can only be studied in the cellular context.

Multicolor fluorescent *in situ* hybridization (FISH) enabled the simultaneous visualization of multiple DNA sequences in fixed cells and indicated the territorial organization of all chromosomes in interphase nuclei (1). In general, the subnuclear distribution of chromosomal segments within the nucleus and with respect to chromosome territories seems to correlate with their gene density and transcriptional activity (2–4). Besides these general principles of genome organization, there is by now good evidence for spatial (re-)organization of the genome during differentiation (5). These changes in genome organization during cellular differentiation might be caused by changes in transcriptional activity, DNA and histone modifications as well as altered proteome composition. For example, the dramatic genome reorganization during myogenesis was linked to the expression of methylcytosine binding proteins (6). Likewise, developmental expression patterns of the nuclear envelope proteins Lamin A/C (LamA/C) and the Lamin B receptor (LBR) control peripheral tethering of facultative heterochromatin and gene expression patterns (7). To what extent and in which cases the relative nuclear position of genes is cause or consequence of transcriptional activity remains to be clarified.

One challenge in addressing these basic questions is the temporal resolution, as changes in genome organization might be fast and transient. To study the dynamics of chromosomal loci, Lac operator repeats were inserted and traced with Lac repressor green fluorescent protein (GFP) fusion proteins (8). With this genetic tag, rapid movements of a DNA chromosome region were observed in response to gene activation (9,10). However, this method is limited to artificially inserted bacterial DNA sequences and thus not applicable to native endogenous DNA sequences. Alternatively, chromosome dynamics in general can be monitored with histone GFP fusions (11), but with this approach, specific DNA sequences cannot be distinguished.

A well-established technology to create recombinant specific DNA binding modules is based on the Cys_2_His_2_ zinc finger (ZF) domains and their 3-bp DNA recognition code (12–14). These domains can be combined to polydactyl zinc finger proteins (PZF) that bind user-defined DNA target sequences. PZF have been used for tracing and manipulating specific DNA sequences *in vivo* (15,16) as well as for gene activation and genome engineering (17–21). Nevertheless, PFZ target choice is biased toward GC-rich sequences, and fusion of individual ZF modules can influence their individual binding specificity, making the generation of PZF for a desired sequence a laborious and cost-intensive process (22).

However, in the past years, a new technology has emerged that overcomes several of the limitations associated with the use of PZF as artificial DNA binding domains. Transcription activator-like effector proteins (TALEs) from the plant pathogen genus *Xanthomonas* contain a DNA binding domain that can be adjusted to bind any desired target sequence with high specificity (23–27). The central TALE DNA binding domain is composed of tandem arranged 33–35 amino acid repeats, with each repeat binding to one base. The base preference of the individual repeats is specified by amino acids 12 and 13, referred to as repeat variable diresidues (RVDs) (28,29). This straightforward correlation between RVD and bound nucleotide allows the fast and efficient generation of DNA binding modules for any user-defined target sequence leading to a broad application of designer TALEs (dTALEs) in genome engineering and as artificial transcription factors (23–27,30–32).

Here we describe the application of dTALEs as a tool for targeting and tracing of repetitive DNA sequences in living cells. We show that dTALEs can be used to visualize the dynamics of major satellite repeats in mouse embryonic stem cells (ESCs) throughout the cell cycle and to characterize their *in vivo* binding kinetics.

## MATERIALS AND METHODS

### Plasmid construction

H2B C-terminally fused to monomeric red fluorescent protein (H2B-mRFP) was described previously (33). The coding sequences of Cbx1 (NM_007622.3) and Cbx 5 (NM_007626.3) were amplified by polymerase chain reaction from pGST-Cbx1 (34) and wild-type E14 cDNA (35), respectively, placed under the control of the cytomegalovirus (CMV) promoter, and fused with mRFP by replacing the ligase I cDNA in the pCMV-mRFP-ligase I plasmid described previously (36). *TALE* genes were cloned in pEXPR IBA3 (IBA, Göttingen) by BsaI cut-ligation (enhanced GFP (eGFP), TALE N/C-terminal regions) and BpiI cut-ligation (TALE DNA binding domain), respectively. Therefore, the BpiI restriction site of pEXPR IBA3 was removed by site-directed mutagenesis using primers P1 and P2. Furthermore, the 3' BsaI overlap of the multiple cloning site was changed from GCGC to AAGG by site-directed mutagenesis using primers P3 and P4. eGFP (27), TALE N- and C-terminal regions were amplified with primers P5 and P6, P7 and P8, and P9 and P10, respectively. Thereby BsaI restriction sites and appropriate overlaps were added and the parts were subsequently assembled in pEXPR IBA3 by BsaI cut-ligation resulting in pEXPR IBA3 eGFP TALE N/C. The DNA binding domain was assembled as described in (24) and cloned via BpiI cut-ligation into the pre-assembled pEXPR IBA3 eGFP TALE N/C to generate the GFP-msTALE.

P1 pEXPR-IBA3 BpiI* F
GGATTGGGAAGATAATAGCAGGCATGC
P2 pEXPR-IBA3 BpiI* R
GCATGCCTGCTATTATCTTCCCAATCC
P3 pEXPR-IBA3 BsaI GCGC-AAGG F
CCATGGTCTCAAAGGTTGGAGCCACCCGC
P4 pEXPR-IBA3 BsaI GCGC-AAGG R
GCGGGTGGCTCCAACCTTTGAGACCATGG
P5 GFP F
TTTGGTCTCTAATGGTGAGCAAG
P6 GFP R
GTCTCAGGTGAAATCGCCCAT
P7 AvrBs3 N-term BsaI F
TTTGGTCTCTCACCATGGATCCCATTCGTTCGCG CAC
P8 AvrBs3 N-term BsaI ATAA R
AAAGGTCTCATTATGGGAAGACCGCGTAAGGT TCAGG
P9 AvrBs3 C-term BsaI ATAA F
TTTGGTCTCTATAAGGGAAGACGGCGCTGGAG
P10 AvrBs3 C-term (till BamHI) BsaI AAGG R
TTTGGTCTCCCTTAGGATCCGGGAGGCCGCCCC

### Cell culture, transfection and fluorescence-activated cell sorting

HEK 293T cells were cultured and transfected as described before (27). J1 ESCs (37) were maintained on gelatin-coated dishes in Dulbecco’s modified Eagle’s medium supplemented with 16% fetal bovine serum (Biochrom), 0.1 mM ß-mercaptoethanol (Invitrogen), 2 mM l-glutamine, 1× MEM non-essential amino acids, 100 U/ml penicillin, 100 µg/ml streptomycin (PAA Laboratories GmbH), 1000 U/ml recombinant mouse LIF (Millipore), 1 µM PD032591 and 3 µM CHIR99021 [Axon Medchem, (38)]. Transfections in ESCs were performed using Lipofectamin 2000 (Invitrogen) according to the manufacturer’s instructions. Fluorescence-activated cell sorting (FACS) was performed with an FACS Aria II (Becton Dickinson).

### Generation of transgenic cell lines

ESCs stably carrying the GFP-msTALE construct were generated by transfecting wt J1 ESCs (37) followed by G418 antibiotic selection (750 µg/ml) and repeated sorting for eGFP expression. To obtain clonal transgenic cell lines, single cell sorting was performed. Single cell clones were analyzed by high content imaging using the Operetta system (PerkinElmer). 4′,6-diamidino-2-phenylindole (DAPI) and eGFP fusion proteins were excited, and the emission was recorded using standard filter sets and 200 ms exposure. For each well, nine different fields were imaged and analyzed with the Harmony analysis software. Double transgenic cell lines were generated by transfecting the stable GFP-msTALE cell line with mRFP-PCNA and H2B-mRFP followed by repeated sorting for eGFP and RFP expression.

### Immunoprecipitation and western blot

Immunoprecipitation was performed as described before (39). One p100 of HEK293T cells transiently transfected with the GFP-msTALE fusion protein or stable GFP-msTALE ESCs, respectively, was harvested and lysed. GFP fusions were pulled down using the GFP-Trap (40) (Chromotek) and subjected to western blotting using a mouse monoclonal anti-GFP antibody (Roche, 11814460001). For comparison of protein levels, stable GFP-msTALE ESCs were lysed in Radio-Immunoprecipitation Assay (RIPA) buffer. Lysate from 750 000 cells was subjected to western blotting using a mouse monoclonal anti-GFP (Roche, 11814460001), rabbit polyclonal anti-CBX1 (Abcam, ab10478) and rabbit polyclonal anti-CENPB (Abcam, ab25743).

### Immunofluorescence staining and microscopy

Immunostaining and 3D-FISH were performed as described previously (41). Briefly, cells cultured on coverslips were fixed with 4% paraformaldehyde for 10 min, washed with PBST (PBS, 0.01% Tween20) and permeabilized with 0.5% Triton X-100. Both primary and secondary antibodies were diluted in blocking solution (PBST, 4% bovine serum albumin). Coverslips with cells were incubated with primary and secondary antibody solutions in dark humid chambers for 1–2 h at room temperature; washings after primary and secondary antibodies were done with PBST. For immuno-FISH, both primary (anti-GFP) and secondary antibodies were applied first; subsequently, cells were postfixed with 4% paraformaldehyde and pre-treated for hybridization. Hybridization was carried out for 2 days at 37°C; posthybridization washings included 2 × Saline Sodium Citrate (SSC) at 37°C and 0.1 × SSC at 61°C (41). The probe for major satellite repeats was generated by polymerase chain reaction using mouse Cot1 DNA (primers: 5′-GCG AGA AAA CTG AAA ATC AC and 5'-TCA AGT CGT CAA GTG GAT G), labeled with Cy3-dUTP by nick-translation, and dissolved in hybridization mixture (50% formamide, 10% dextran sulfate, 1 × SSC) at a concentration of 10–20 ng/µl. For nuclear DNA counterstaining, DAPI was added to the secondary antibody solution to the final concentration 2 µg/ml. Coverslips were mounted in antifade medium (Vectashield, Vector Laboratories) and sealed with colorless nail polish.

Following primary antibodies were used: anti-GFP (Roche, 11814460001), anti-lamin B1 (Santa Cruz Biotechnology, sc-6217), anti-nucleophosmin (B23, Sigma-Aldrich, B0556), anti-kinetochores (Euroimmun AG, CA 1611-0101) and anti-H4K20me3 (Abcam, ab9053). The secondary antibodies were anti-rabbit conjugated to DyLight fluorophore 594 (Jackson ImmunoResearch, 711-505-152), anti-mouse conjugated to Alexa 488 and 555 (Invitrogen, A21202 and A31570), anti-goat conjugated to Cy3 (Jackson ImmunoResearch, 706-166-148) or Alexa 647 (Invitrogen, A21447) and anti-human conjugated to Cy3 (Jackson ImmunoResearch, 309-165-003). Single optical sections or stacks of optical sections were collected using a Leica TCS SP5 confocal microscope equipped with Plan Apo 63×/1.4 NA oil immersion objective and lasers with excitation lines 405, 488, 561 and 633 nm. Dedicated plug-ins in ImageJ program were used to compensate for axial chromatic shift between fluorochromes in confocal stacks, to create RGB images/stacks and to arrange them into galleries (42,43).

### Live cell microscopy, fluorescence loss in photobleaching and quantitative fluorescence recovery after photobleaching analysis

Live cell imaging, fluorescence loss in photobleaching (FLIP) and fluorescence recovery after photobleaching (FRAP) experiments were performed on an Ultra*VIEW* VoX spinning disc microscope (PerkinElmer) as described before (44). Photobleaching was performed using two iterations with the acousto-optic tunable filter (AOTF) of the 488 nm laser line set to 100% transmission. For acquisition of FRAP or FLIP experiments, the 488 nm laser line was set to 20% transmission.

In FRAP experiments, a circular bleach region of 2.5 × 2.5 µm, covering one chromocenter (CC) per cell, was chosen. After 20 prebleach frames with a time interval of 200 ms, CCs in five cells were bleached (∼650 ms). Then 150 postbleach frames were recorded with a time interval of 200 ms followed by 800 postbleach frames with a time interval of 500 ms. The mean intensity of this circular region was measured over time. Data correction, double normalization and calculation of the half time recovery (*t_1/2_*) and the mobile fraction (*MF*) were performed as described before (45). The outline of the nucleus for the evaluation was determined using images obtained with bright field illumination. The results of 14 cells were averaged.

In FLIP experiments, approximately half of the cell was bleached in a rectangular region. Like in the FRAP experiments, multiple cells were bleached in parallel. After initial five prebleach frames with a time interval of 4 s, 40 bleach cycles were performed. Each bleach cycle consisted of a bleaching event of ∼700 ms followed by 10 time frames with a time interval of 4 s. In each frame, a z-stack of 7 µm with a step size of 1 µm was recorded to check for axial drift. To evaluate the data, the mean intensity of a circular region of 20 × 20 pixel was determined over time. The region covered either a CC in the unbleached half of a cell, in which the other half was repeatedly bleached [Figure 3B (1)], or in a neighboring unbleached cell [Figure 3B (2)]. Afterward, the background was subtracted from these results. The measurements were performed in Fiji (46) followed by calculations in Excel.

In long-term imaging experiments, a z-stack of 10.8–14.4 µm with a step size of 1.2 µm was recorded every 15 min for ∼20 h. To avoid photodamage of the cells, the AOTF of the laser was set to low transmission values of 6–10%.

## RESULTS AND DISCUSSION

### Generation of a GFP-msTALE highlighting major satellite DNA in mouse cells

**Figure 1.**
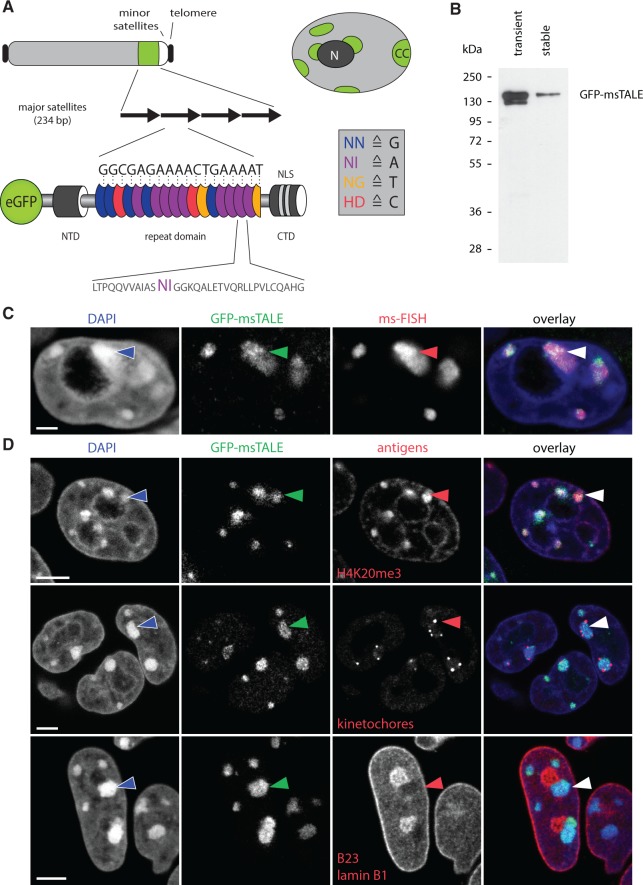
Localization of the GFP-msTALE to major satellite repeats in mouse pericentromeric heterochromatin. (**A**, top) Schematic representation of an acrocentric mouse chromosome with telomeres (black), major satellites (green), minor satellites (white) and the long arm of the chromosome (light gray). Overview of a nucleus showing multiple heterochromatin centers (CC, green), where the major satellite DNA is clustered. CCs localize next to the nuclear periphery and the nucleoli (dark gray, N) and are surrounded by less condensed chromatin (light gray). (A, bottom) Schematic representation of the GFP-msTALE aligned to its binding site within the major satellite repeats (black arrows). The dTALE is composed of an N-terminal domain (NTD), a C-terminal domain (CTD) bearing nuclear localization signals (NLS) and a central repeat domain. DNA target recognition is mediated by the RVDs within each TALE repeat (blue, purple, yellow and red ellipses for RVDs binding to the bases G, A, T and C, respectively, single letter code for amino acids and nucleotide bases). A representative repeat sequence with RVDs (purple) is shown below as close-up (single letter code for amino acids). The complete sequence is shown in Supplementary Figure S1. Note that the msTALE is lacking the C-terminal activation domain. For visualization and immunoprecipitation, the dTALE is N-terminally fused to GFP. (**B**) GFP-Trap pull-down from HEK293T cells transiently transfected with the GFP-msTALE construct (transient) and a J1 ESC clone stably expressing the GFP-msTALE (stable). Immunodetection by an anti-GFP antibody. (**C**) 3D-immuno-FISH on stable GFP-msTALE ESCs with probe directed against major satellite repeats (ms-FISH). Because the GFP signal is strongly reduced by 3D-FISH procedure, an anti-GFP antibody was used to visualize GFP-msTALE localization. Note strict co-localization of the GFP signal (green) and the FISH probe (red). Nuclei were counterstained with DAPI (blue). Arrowhead points at one of the CCs. (**D**) Immunostaining of ESCs stably expressing the GFP-msTALE (green). Upper panel, antibodies against heterochromatin (anti-H4K20me3, red) mark GFP-positive CCs. Middle panel, human antiserum binding to kinetochores reveals kinetochore clusters (red) at the surface of CCs. Lower panel, CCs marked with GFP-msTALE show a characteristic intranuclear localization abutting nuclear periphery or adjacent to the nucleoli (both shown in red). Nuclei were counterstained with DAPI (blue). Arrowheads mark one of the CCs in each exemplified nucleus. All images are single optical confocal sections. Scale bars: C, 5 µm; D, 2 µm.

To test whether dTALEs are suitable to trace DNA sequences *in vivo*, we generated an N-terminal GFP fusion construct directed against the 19-bp sequence 5'-TGGCGAGAAAACTGAAAAT-3' of the murine major satellite repeat sequence using a golden-gate cloning-based approach (Supplementary Figure S1) (24). The 234-bp units of the major satellite repeat are present in 1000–10 000 copies per chromosome located in the centromeric periphery (47). Major satellite repeats constitute the major part of mouse CCs. These heterochromatin regions are clustered centromeres of acrocentric chromosomes located at the nuclear periphery and around the nucleoli (Figure 1A) (48). The distinct subnuclear localization and high copy number of major satellite repeats constitute an ideal model system to test the applicability of dTALEs for *in vivo* tracing of DNA sequences.

Using the dTALE directed against the major satellite repeat (GFP-msTALE), we generated a stable mouse ESC line by antibiotic selection followed by repeated FACS sorting. After single cell sorting, we established a clone that exhibited correct protein size of the GFP-msTALE compared with a transient transfection in HEK293T cells (Figure 1B). The cell line expressed relatively low levels of the GFP-msTALE compared with the endogenous heterochromatin-associated protein Cbx1 and the kinetochore binding protein CenpB (Supplementary Figure S2). To address the specificity of the TALE binding to the major satellite repeats, we performed fluorescence *in situ* hybridization on 3D-preserved cells (3D-FISH) (Figure 1C). Using a directly labeled probe directed against the major satellites combined with immunostaining, we observed strict co-localization of the GFP-msTALE with the major satellite foci. To further characterize the stable ESC line, the subnuclear localization of the GFP-msTALE in relation to several makers of nuclear structures was assessed. In interphase cells, the GFP signal exhibited a focal pattern co-localizing with DAPI-stained CCs as well as with trimethylated histone 4 lysine 20 (H4K20me3), a marker for constitutive heterochromatin highly enriched in major satellite repeats (46) (Figure 1D, upper panel). Furthermore, we found that kinetochores are localized in the periphery of the GFP foci consistent with the expected relative organization of CCs (Figure 1D, middle panel). Immunolabeling against nucleophosmin (B23), a marker enriched in nucleoli and lamin B1, revealed that the GFP foci localize around the nucleoli and in the nuclear periphery (Figure 1D, lower panel). For direct comparison, we transiently expressed two heterochromatin proteins (RFP-Cbx1 and RFP-Cbx5, also known as heterochromatin protein 1 beta and alpha), which co-localized with the GFP-msTALE at CCs (Supplementary Figure S3A and B) until G2 phase, when binding of both Cbx1 and Cbx5 is abolished (49). Taken together, these data demonstrate that the GFP-msTALE correctly highlights the localization of the major satellite DNA *in vivo.*

### The GFP-msTALE enables DNA sequence tracing during the cell cycle *in vivo*

**Figure 2.**
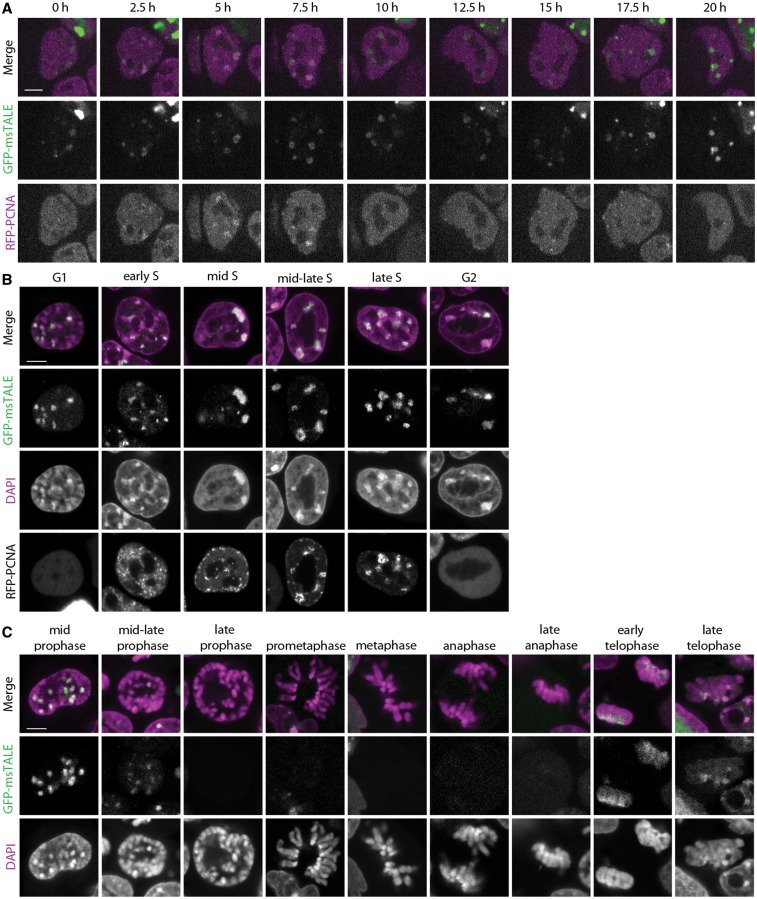
Cell cycle-dependent distribution of GFP-msTALE. (**A**) Live cell imaging of replicating stable GFP-msTALE cell line (green) stably transfected with RFP-PCNA (magenta). (**B**) Single confocal sections of fixed and RFP-PCNA co-transfected GFP-msTALE stable cell line (green) during DNA replication. DNA is visualized by DAPI (magenta). (**C**) Single confocal sections of fixed GFP-msTALE cell line (green) during mitosis. DNA is visualized by DAPI (magenta). Scale bars: 5 μm.

After establishing the correct localization of the GFP-msTALE, we analyzed its behavior during cell cycle progression. We generated double transgenic cell lines also expressing H2B-RFP (Supplementary Figure S3C) to visualize whole chromatin in combination with GFP-msTALE-bound CCs. To distinguish the different phases of DNA replication, we stably transfected the GFP-msTALE ESC line with the S-phase marker RFP-PCNA (50) and acquired time series over 20 h demonstrating the suitability of the approach for live cell imaging. We observed progression throughout S-phase with the GFP-msTALE exhibiting the typical focal pattern expected for major satellite DNA (Figure 2A, Supplementary Movie S1). Importantly, the GFP-msTALE was located at mid to late S-phase replication foci, correlating well with the replication of CCs (45,50) (Figure 2A and B).

Next, we analyzed the localization of the GFP-msTALE through mitosis. Although the dTALE still localized to chromosomes until mid prophase, it largely dissociated in mid to late prophase and reassociated in early telophase (Figure 2C). Residual binding in metaphase was visible on contrast enhancement and with higher expression levels (Supplementary Figure S3). It should be noted that while this work was under review, a smaller TALE construct targeting another major satellite repeat sequence was described that exhibited more stable DNA binding throughout mitosis (51). These results indicate that the chromatin condensation during mitosis might affect binding of the dTALE to its target sequences, an observation in line with the hypothesis that chromatin environment can influence dTALE binding and activity (27,52).

### Analysis of *in vivo* protein dynamics reveals a strong but dynamic association of the GFP-msTALE with CCs

**Figure 3.**
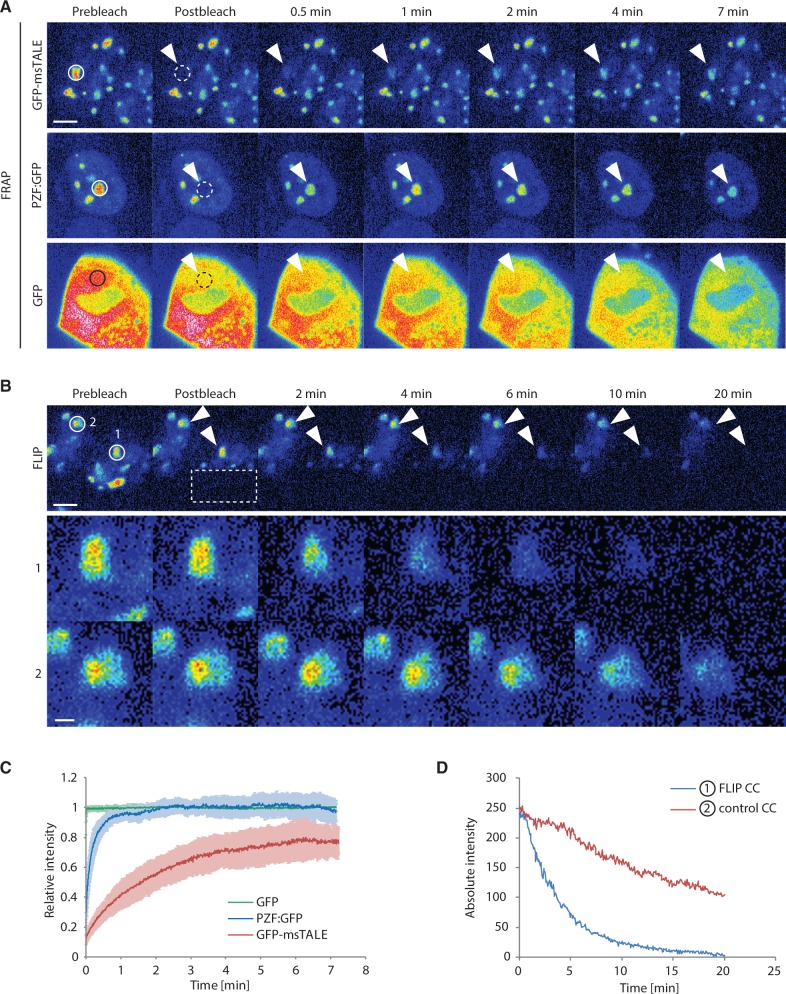
Comparative analysis of the dynamics of GFP, GFP-msTALE and PZF:GFP by FRAP and FLIP. Continuous lines indicate the intensity measurement areas, whereas dashed lines indicate the bleached regions. The arrowheads point to the intensity measurement areas in the postbleach time points. These regions are magnified by a factor of four in the lower panel of (B). Scale bars: 5 µm (upper panel) and 1 µm (lower panel, (B)). (**A**) Representative FRAP experiment for GFP, the stable GFP-msTALE cell line and PZF:GFP. A circular region (dashed line) with a diameter of 2.5 µm was bleached. (**B**) Representative FLIP experiment of the GFP-msTALE. A rectangular region indicated by the dashed line was repeatedly bleached. CCs in the unbleached half of the bleached cell (1) and in an unbleached reference cell (2) are highlighted. (**C**) Quantitative evaluation of FRAP experiments (average of 12–14 cells) comparing GFP-msTALE, PZF:GFP and GFP. Error bars represent standard deviation. (**D**) Representative background corrected, absolute intensities of two CCs in a bleached cell (1, blue line) and an unbleached reference cell (2, red line) illustrated in (B).

The observation that the dTALE is not associated with condensed chromosomes during mitosis prompted us to investigate the *in vivo* DNA binding kinetics of the GFP-msTALE in more detail. To quantify the binding dynamics of the GFP-dTALE in living cells, we performed FRAP and FLIP experiments. Both methods were used in a complementary approach (53,54), with intensity measurements focusing on single CCs, the prominent binding sites of the GFP-msTALE. For the FRAP experiment, we bleached a small circular region including one CC and quantified the recovery in this region over time (Figure 3A and C). The faster the recovery, the more transient is the binding dynamics. In contrast, for the FLIP experiment we bleached half of the nucleus repeatedly and quantified the intensity of a CC in the unbleached half (Figure 3B and D). On protein dissociation from the binding site in the unbleached region and diffusion into the bleached region, a reduction of signal intensity in the unbleached half of the nucleus can be observed. The faster the signal decreases, the more transient is the binding dynamics. Thus, the binding dynamics of GFP-dTALEs can be analyzed without inducing damage at these sites by photobleaching. As the bleached regions and the measured region are separate from each other, this approach also takes into account the mobility between different compartments (55,56).

In our quantitative FRAP experiments with the GFP-msTALE, we observed a gradual slow increase in intensity over 7 min (Figure 3C). Even after this time, an immobile, not yet recovered fraction of ∼20% was still detectable, indicating a strong binding of the dTALE to the major satellites. Unlike freely diffusing proteins such as GFP (44), no fast initial recovery of GFP-msTALE was detected in the bleached area (Figure 3A). Also the signal intensity outside CCs was rather low, indicating that most of the GFP-msTALE was bound to chromosomes. The stable interaction of GFP-msTALE with major satellites DNA seen in the FRAP experiments becomes directly evident from the complementary FLIP experiments. Even on repeated bleaching, GFP-msTALE fluorescence was detectable at the unbleached CCs within the same nucleus for up to 20 min (Figure 3D, line 1). Notably, reference measurement of CCs in the adjacent cell (Figure 3D, line 2) revealed a continuous loss of fluorescence (∼50% over 20 min) because of image acquisition with these microscope settings, so that the actual dissociation of GFP-msTALE from CCs (Figure 3D, line 1) is even slightly slower. For comparison, we tested the ZF-based PZF:GFP binding to the major satellite repeats (15) and obtained a fast initial recovery together with a 10 times lower half time recovery value in FRAP experiments as well as a faster loss of fluorescence in FLIP experiments (Figure 3A and C and Supplementary Figure 4B) exhibiting less stable binding than the TALE construct.

*In vitro* studies revealed that dTALEs have *K_d_* values in the low nanomolar to high picomolar range (27,57), indicating a strong binding affinity of dTALEs to DNA. Our *in vivo* binding studies demonstrate that dTALEs also strongly bind their target DNA sequence in a chromatin context, but not in a static mode, and reveal a rather dynamic interplay with chromatin. The depletion of GFP-msTALE from highly condensed chromatin during mitosis could be due to conformational changes of the DNA substrate weakening the binding or preventing rebinding that shifts the dynamic equilibrium toward the unbound state. This is consistent with the recent observation that condensation may affect binding and access of nuclear proteins to chromatin (33).

In summary, we could show that dTALEs can be engineered to bind and highlight repetitive DNA sequences *in vivo*. With the example of a dTALE designed to target the major satellite repeats in mouse cells, we showed that fluorescent dTALEs are suitable for live cell imaging of specific DNA sequences throughout the cell cycle. Moreover, we found that dTALEs can detect changes in chromatin condensation and that a dynamic interplay between dTALE and chromatin exists. In this study, we targeted repetitive DNA sequences; with more sensitive detection as used in single molecule tracing setups, eventually single copy genes might also become traceable.

Fluorescently labeled dTALEs open new perspectives to trace specific endogenous DNA sequences at high temporal and spatial resolution in living cells. Strict sequence dependent localization, higher affinity and easy assembly of dTALEs give this method an advantage over previous techniques based on overexpression of chromatin binding factors or ZF arrays in tracing and targeting of specific DNA sequences in living cells. This novel application of dTALEs will help to identify and elucidate cell cycle and development-specific changes in genome organization and chromatin dynamics.

## SUPPLEMENTARY DATA

Supplementary Data are available at NAR Online.

## FUNDING

The Deutsche Forschungsgemeinschaft [SFB924 to T.L., SO1054 to I.S. and SFB646 to H.L.]; the Two Blades Foundation (to T.L.); the Nanosystems Initiative Munich [to H.L.]; the Bundesministerium für Bildung und Forschung [EpiSys to H.L.]; The International Max Planck Research School for Molecular and Cellular Life Sciences (IMPRS-LS) (to K.T. and K.S.); The Bundesministerium für Bildung und Forschung [3D-SR, in part to K.T.]. Funding for open access charge: Deutsche Forschungsgemeinschaft.

*Conflict of interest statement*. T.L. is a co-inventor of a patent application regarding the use of TALEs.

## Supplementary Material

Supplementary Data
