# Supplementary material for: Targeting and tracing of specific DNA sequences with dTALEs in living cells
Source: Nucleic Acids Res. 2013 Dec 25;42(6):e38. doi: 10.1093/nar/gkt1348 (PMC3973286; doi:10.1093/nar/gkt1348)
Supplement: Supplementary Data [file supp_42_6_e38__index.html]

Targeting and tracing of specific DNA sequences with dTALEs in living cells — Supplementary Data 

# Targeting and tracing of specific DNA sequences with dTALEs in living cells

## Supplementary Data

files

**Files in this Data Supplement:**

- Supplementary Data - pdf file
- Supplementary Data - avi file
