# Supplementary material for: Targeting and tracing of specific DNA sequences with dTALEs in living cells
Source: Nucleic Acids Res. 2013 Dec 25;42(6):e38. doi: 10.1093/nar/gkt1348 (PMC3973286; doi:10.1093/nar/gkt1348)
Supplement: Supplementary Data [file supp_gkt1348_nar-02554-met-k-2013-File006.pdf]

Supplementary Figure 1: Nucleotide and amino acid sequence of the GFP-msTALE. The entire open reading frame of the fusion protein is shown: GFP is highlighted in green, repeat divariable residues (RVD) within each TALE repeat are highlighted in blue, purple, yellow and red for RVDs binding to the bases G, A, T and C, respectively. Single letter code is used for amino acids and nucleotide bases.

Supplementary Movie 1: Cell-cycle dependent distribution of GFP-msTALE. Live cell imaging of replicating stable GFP-msTALE cell line (green) cotransfected with RFP-PCNA (magenta).

Supplementary Figure 2: Expression of the stably integrated GFP-msTALE construct is relatively low compared to endogenous protein levels of major satellite associated proteins. Western blot showing protein levels of the GFP-msTALE, CenpB and Cbx1 in the stable GFP-msTALE ES cell line.

Supplementary Figure 3: Live cell imaging of GFP-msTALE together with chromatin associated proteins (A) Live cell imaging of replicating stable GFP-msTALE cell line (green) cotransfected with RFP-Cbx1 (magenta). (B) Live cell imaging of replicating stable GFP-msTALE cell line (green) cotransfected with RFP-Cbx5 (magenta). (C) Live cell imaging of replicating stable GFP-msTALE cell line (green) stably transfected with H2B-RFP (magenta). Arrowheads point towards one representative chromocenter. Scale bars: 5 µm.

Supplementary Figure 4: Comparative analysis of the dynamics of the GFP-msTALE , the PZF:GFP and GFP. (A) Representative FLIP experiments of the stable GFP-msTALE cell line and PZF:GFP (FLIP of the GFP-msTALE taken from Figure 3B for direct comparison). A rectangular region indicated by the dashed line was repeatedly bleached. Chromocenters in the unbleached half of the bleached cell (1) and in an unbleached reference cell (2) are highlighted. Continuous lines indicate the intensity measurement areas, whereas dashed lines indicate the bleached regions. Arrowheads point to the intensity measurement areas in the postbleach time points. These regions are magnified by a factor of four in the lower panels for both stable GFP-msTALE ESC line and the PZF:GFP. Scale bars: 5 µm (upper panels), 1 µm (lower panels). (B) Kinetic properties of GFP, GFP-msTALE and PZF:GFP analyzed by FRAP. N indicates the number of analyzed cells, *MF* the mobile fraction and $t_{1/2}$ the half time of recovery. Mean values ± standard deviation are listed.

Supplementary Figure 1

```
ATGGTGAGCAAGGGCGAGGAGCTGTTCACCGGGGTGGTGCCCATCCTGGTCGAGCTGGACGGCGACGTAAACGGCCACAAGTTCAGCGTGTCCGGCGAGGGCGAGGGCGATGCCACCTAC
M V S K G E E L F T G V V P I L V E L D G D V N G H K F S V S G E G E G D A T Y

GGCAAGCTGACCCTGAAGTTCATCTGCACCACCGGCAAGCTGCCCGTGCCCTGGCCCACCCTCGTGACCACCCTGACCTACGGCGTGCAGTGCTTCAGCCGCTACCCCGACCACATGAAG
G K L T L K F I C T T G K L P V P W P T L V T T L T Y G V Q C F S R Y P D H M K

CAGCACGACTTCTTCAAGTCCGCCATGCCCGAAGGCTACGTCCAGGAGCGCACCATCTTCTTCAAGGACGACGGCAACTACAAGACCCGCGCCGAGGTGAAGTTCGAGGGCGACACCCTG
Q H D F F K S A M P E G Y V Q E R T I F F K D D G N Y K T R A E V K F E G D T L

GTGAACCGCATCGAGCTGAAGGGCATCGACTTCAAGGAGGACGGCAACATCCTGGGGCACAAGCTGGAGTACAACTACAACAGCCACAACGTCTATATCATGGCCGACAAGCAGAAGAAC
V N R I E L K G I D F K E D G N I L G H K L E Y N Y N S H N V Y I M A D K Q K N

GGCATCAAGGTGAACTTCAAGATCCGCCACAACATCGAGGACGGCAGCGTGCAGCTCGCCGACCACTACCAGCAGAACACCCCCATCGGCGACGGCCCCGTGCTGCTGCCCGACAACCAC
G I K V N F K I R H N I E D G S V Q L A D H Y Q Q N T P I G D G P V L L P D N H

TACCTGAGCACCCAGTCCGCCCTGAGCAAAGACCCCAACGAGAAGCGCGATCACATGGTCCTGCTGGAGTTCGTGACCGCCGCCGGGATCACTCTCGGCATGGACGAGCTGTACAAGAGC
Y L S T Q S A L S K D P N E K R D H M V L L E F V T A A G I T L G M D E L Y K S

GGCCTGAGGAGCAGAGCCCAGGCGAGCAACAGCGCCGTGGACGCCACCATGGGCGATTTCACCATGGATCCCATTCGTTCGCGCACACCAAGTCCTGCCCGCGAGCTTCTGCCCGGACCC
G L R S R A Q A S N S A V D A T M G D F T M D P I R S R T P S P A R E L L P G P

CAACCCGATGGGGTTCAGCCGACTGCAGATCGTGGGGTGTCTCCGCCTGCCGGCGGCCCCCTGGATGGCTTGCCCGCTCGGCGGACGATGTCCCGGACCCGGCTGCCATCTCCCCCTGCC
Q P D G V Q P T A D R G V S P P A G G P L D G L P A R R T M S R T R L P S P P A

CCCTCACCTGCGTTCTCGGCGGGCAGCTTCAGTGACCTGTTACGTCAGTTCGATCCGTCACTTTTTAATACATCGCTTTTTGATTCATTGCCTCCCTTCGGCGCTCACCATACAGAGGCT
P S P A F S A G S F S D L L R Q F D P S L F N T S L F D S L P P F G A H H T E A

GCCACAGGCGAGTGGGATGAGGTGCAATCGGGTCTGCGGGCAGCCGACGCCCCCCCACCCACCATGCGCGTGGCTGTCACTGCCGCGCGGCCGCCGCGCGCCAAGCCGGCGCCGCGACGA
A T G E W D E V Q S G L R A A D A P P P T M R V A V T A A R P P R A K P A P R R

CGTGCTGCGCAACCCTCCGACGCTTCGCCGGCCGCGCAGGTGGATCTACGCACGCTCGGCTACAGCCAGCAGCAACAGGAGAAGATCAAACCGAAGGTTCGTTCGACAGTGGCGCAGCAC
R A A Q P S D A S P A A Q V D L R T L G Y S Q Q Q Q E K I K P K V R S T V A Q H

CACGAGGCACTGGTCGGCCATGGGTTTACACACGCGCACATCGTTGCGCTCAGCCAACACCCGGCAGCGTTAGGGACCGTCGCTGTCAAGTATCAGGACATGATCGCAGCGTTGCCAGAG
H E A L V G H G F T H A H I V A L S Q H P A A L G T V A V K Y Q D M I A A L P E

GCGACACACGAAGCGATCGTTGGCGTCGGCAAACAGTGGTCCGGCGCACGCGCTCTGGAGGCCTTGCTCACGGTGGCGGGAGAGTTGAGAGGTCCACCGTTACAGTTGGACACAGGCCAA
A T H E A I V G V G K Q W S G A R A L E A L L T V A G E L R G P P L Q L D T G Q

CTTCTCAAGATTGCAAAACGTGGCGGCGTGACCGCAGTGGAGGCAGTGCATGCATGGCGCAATGCACTGACGGGTGCCCCCCTGAACCTTACGCCGCAGCAGGTGGTGGCCATCGCCAGC
L L K I A K R G G V T A V E A V H A W R N A L T G A P L N L T P Q Q V V A I A S

AATAATGGTGGCAAGCAGGCGCTGGAGACGGTGCAGCGGCTGCTTCCGGTGCTGTGCCAGGCCCATGGCCTGACCCCGGAGCAGGTGGTGGCCATCGCCAGCAATAATGGTGGCAAGCAG
N N G G K Q A L E T V Q R L L P V L C Q A H G L T P E Q V V A I A S N N G G K Q

GCGCTGGAGACGGTGCAGCGATTGTTGCCGGTGCTGTGCCAGGCCCATGGCCTGACCCCGGAGCAGGTGGTGGCCATCGCCAGCCACGACGGTGGCAAGCAGGCGCTGGAGACTGTCCAG
A L E T V Q R L L P V L C Q A H G L T P E Q V V A I A S H D G G K Q A L E T V Q

CGGCTGTTGCCGGTGCTGTGCCAGGCCCATGGCCTGACCCCGGAGCAGGTGGTGGCCATCGCCAGCAATAATGGTGGCAAGCAGGCGCTTGAGACGGTGCAGCGGCTGTTGCCGGTGCTG
R L L P V L C Q A H G L T P E Q V V A I A S N N G G K Q A L E T V Q R L L P V L

TGCCAGGCCCATGGCCTGACCCCGGAGCAGGTGGTGGCCATCGCCAGCAATATTGGTGGCAAGCAGGCTCTGGAGACGGTGCAGCGGCTGTTGCCGGTGCTGTGCCAGGCCCATGGCCTG
C Q A H G L T P E Q V V A I A S N I G G K Q A L E T V Q R L L P V L C Q A H G L

ACCCCGGAGCAGGTGGTGGCCATCGCCAGCAATAATGGGGGCAAGCAGGCGCTGGAGACGGTGCAGCGGCTGTTGCCGGTGCTGTGCCAGGCCCATGGCCTGACCCCGCAGCAGGTGGTG
T P E Q V V A I A S N N G G K Q A L E T V Q R L L P V L C Q A H G L T P Q Q V V

GCCATCGCCAGCAATATTGGCGGCAAGCAGGCGCTGGAGACGGTGCAGGCGCTGTTGCCGGTGCTGTGCCAGGCCCATGGCCTGACCCCGGAGCAGGTGGTGGCCATCGCAAGCAATATT
A I A S N I G G K Q A L E T V Q A L L P V L C Q A H G L T P E Q V V A I A S N I

GGTGGCAAGCAGGCGCTGGAGACGGTGCAGGCGCTGTTGCCGGTGCTGTGCCAGGCCCATGGCCTGACCCCGGAGCAGGTGGTGGCAATCGCCAGCAATATTGGTGGCAAGCAGGCGCTG
G G K Q A L E T V Q A L L P V L C Q A H G L T P E Q V V A I A S N I G G K Q A L

GAGACGGTGCAGCGGCTGTTGCCGGTGCTGTGCCAGGCCCATGGCCTGACCCCGCAACAGGTGGTAGCCATCGCCAGCAATATTGGTGGCAAGCAGGCGCTGGAGACGGTGCAGCGGCTG
E T V Q R L L P V L C Q A H G L T P Q Q V V A I A S N I G G K Q A L E T V Q R L

TTGCCGGTGCTGTGCCAGGCCCATGGCCTGACACCCCAGCANGTGGTAGCGATCGCCAGCCACGACGGTGGCAAGCAGGCGCTGGAGACGGTGCAGCGGCTGCTTCCGGTGCTGTGCCAG
L P V L C Q A H G L T P Q X V V A I A S H D G G K Q A L E T V Q R L L P V L C Q

GCCCATGGCCTGACCCCGCAGCAGGTGGTGGCCATCGCCAGCAATGGCGGTGGCAAGCAGGCGCTGGAGACGGTGCAGCGATTGTTGCCGGTGCTGTGCCAGGCCCATGGCCTGACCCCG
A H G L T P Q Q V V A I A S N G G G K Q A L E T V Q R L L P V L C Q A H G L T P

GAGCAGGTGGTGGCCATCGCCAGCAATAATGGTGGCAAGCAGGCGCTGGAGACTGTCCAGCGGCTGTTGCCGGTGCTGTGCCAGGCCCATGGCCTGACCCCGGAGCAGGTGGTGGCCATC
E Q V V A I A S N N G G K Q A L E T V Q R L L P V L C Q A H G L T P E Q V V A I

GCCAGCAATATTGGTGGCAAGCAGGCGCTTGAGACGGTGCAGCGGCTGTTGCCGGTGCTGTGCCAGGCCCATGGCCTGACCCCGGAGCAGGTGGTGGCCATCGCCAGCAATATTGGTGGC
A S N I G G K Q A L E T V Q R L L P V L C Q A H G L T P E Q V V A I A S N I G G

AAGCAGGCTCTGGAGACGGTGCAGCGGCTGTTGCCGGTGCTGTGCCAGGCCCATGGCCTGACCCCGGAGCAGGTGGTGGCCATCGCCAGCAATATTGGGGGCAAGCAGGCGCTGGAGACG
K Q A L E T V Q R L L P V L C Q A H G L T P E Q V V A I A S N I G G K Q A L E T

GTGCAGCGGCTGTTGCCGGTGCTGTGCCAGGCCCATGGCCTGACCCCGCAGCAGGTGGTGGCCATCGCCAGCAATATTGGCGGCAAGCAGGCGCTGGAGACGGTGCAGGCGCTGTTGCCG
V Q R L L P V L C Q A H G L T P Q Q V V A I A S N I G G K Q A L E T V Q A L L P

GTGCTGTGCCAGGCCCATGGCCTGACACCCCAGCAGGTGGTGGCCATCGCCAGCAATGGCGGCGGCAGGCCGGCGCTGGAGAGCATTGTTGCCCAGTTATCTCGCCCTGATCCGGCGTTG
V L C Q A H G L T P Q Q V V A I A S N G G G R P A L E S I V A Q L S R P D P A L

GCCGCGTTGACCAACGACCACCTCGTCGCCTTGGCCTGCCTCGGCGGACGTCCTGCGCTGGATGCAGTGAAAAAGGGATTGCCGCACGCGCCGGCCTTGATCAAAAGAACCAATCGCCGT
A A L T N D H L V A L A C L G G R P A L D A V K K G L P H A P A L I K R T N R R

ATTCCCGAACGCACATCCCATCGCGTTGCCGACCACGCGCAAGTGGTTCGCGTGCTGGGTTTTTTCCAGTGCCACTCCCACCCAGCGCAAGCATTTGATGACGCCATGACGCAGTTCGGG
I P E R T S H R V A D H A Q V V R V L G F F Q C H S H P A Q A F D D A M T Q F G

ATGAGCAGGCACGGGTTGTTACAGCTCTTTCGCAGAGTGGGCGTCACCGAACTCGAAGCCCGCAGTGGAACGCTCCCCCCAGCCTCGCAGCGTTGGGACCGTATCCTCCAGGCATCAGGG
M S R H G L L Q L F R R V G V T E L E A R S G T L P P A S Q R W D R I L Q A S G

ATGAAAAGGGCCAAACCGTCCCCTACTTCAACTCAAACGCCGGATCAGGCGTCTTTGCATGCATTCGCCGATTCGCTGGAGCGTGACCTTGATGCGCCTAGCCCAATGCACGAGGGAGAT
M K R A K P S P T S T Q T P D Q A S L H A F A D S L E R D L D A P S P M H E G D

CAGACGCGGGCAAGCAGCCGTAAACGGTCCCGATCGGATCGTGCTGTCACCGGTCCCTCCGCACAGCAATCGTTCGAGGTGCGCGTTCCCGAACAGCGCGATGCGCTGCATTTGCCCCTC
Q T R A S S R K R S R S D R A V T G P S A Q Q S F E V R V P E Q R D A L H L P L

AGTTGGAGGGTAAAACGCCCGCGTACCAGTATCGGGGGCGGCCTCCCGGATCCTTAA
S W R V K R P R T S I G G G L P D P *
```

Supplementary Figure 2

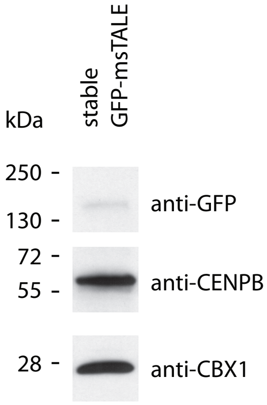

Supplementary Figure 3

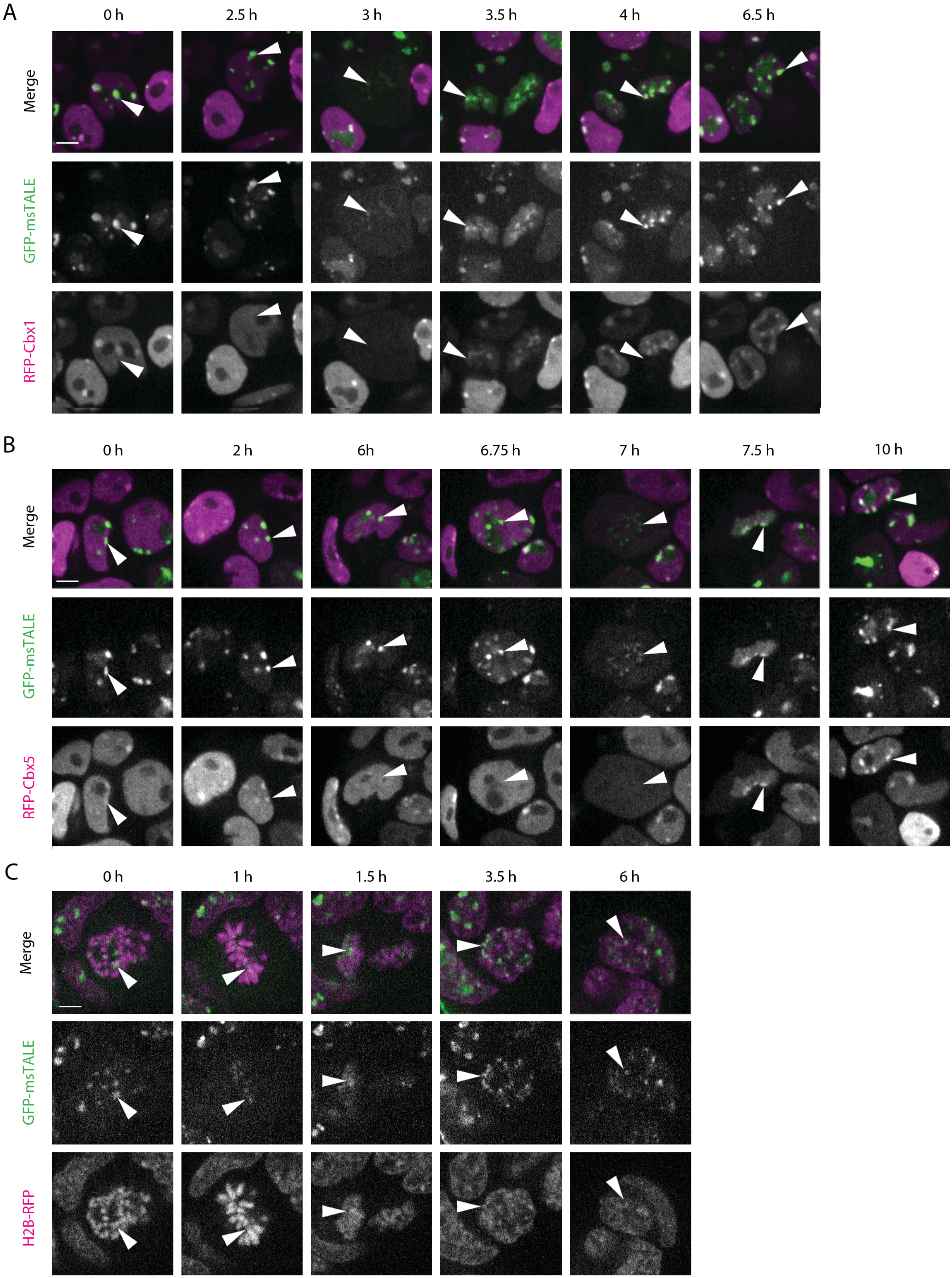

Supplementary Figure 4

A

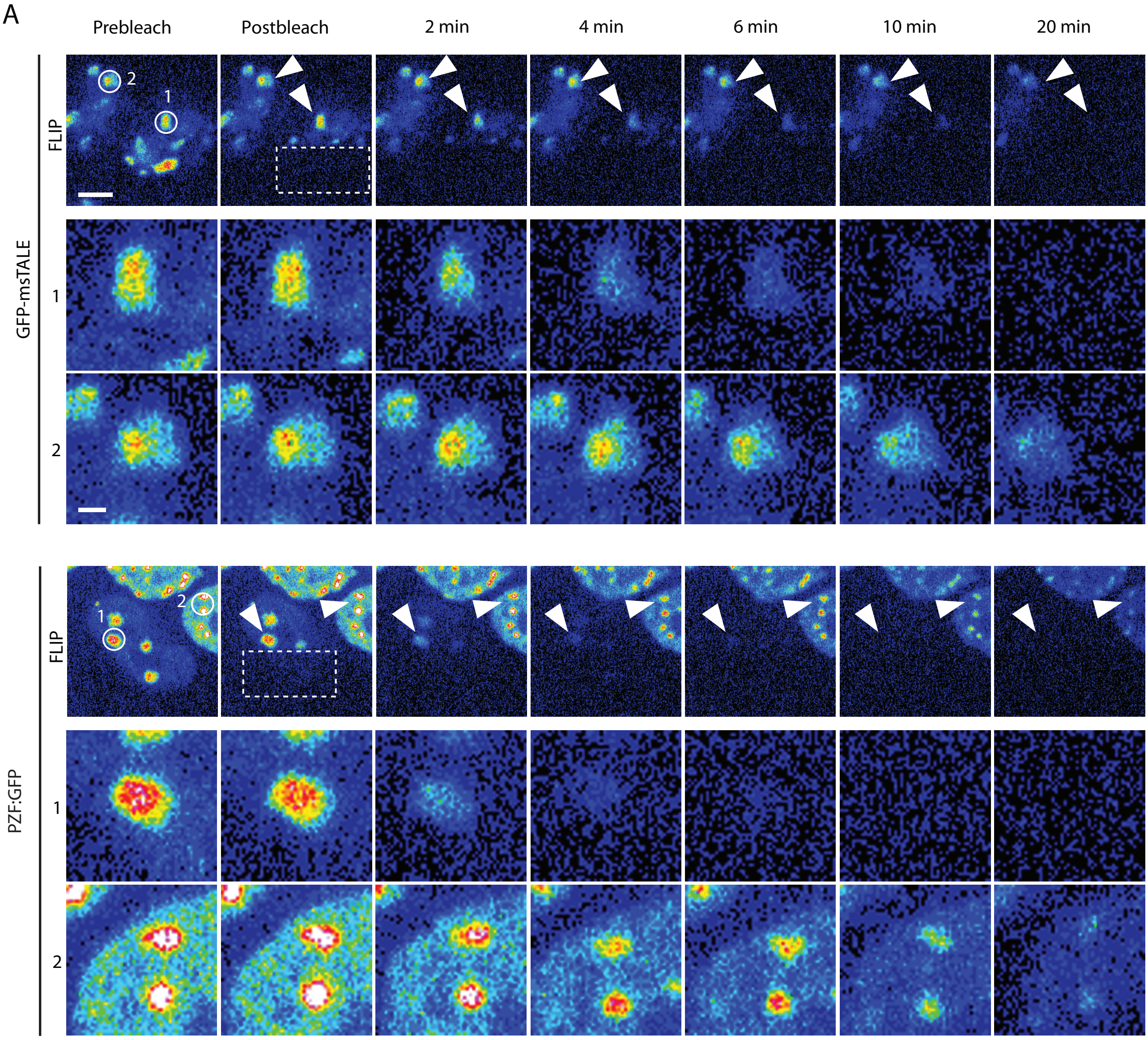

B

	N	*MF* [%] ± SD	$t_{1/2}$[s]
GFP	12	1.0±0.3	0.1±0.1
GFP-msTALE	14	0.7±0.1	77.8±21.2
PZF:GFP	12	1.0±0.1	6.9±3.5
